# Efficacy and Safety of Paromomycin for Visceral Leishmaniasis: A Systematic Review

**DOI:** 10.1155/2021/8629039

**Published:** 2021-07-24

**Authors:** Pashupati Pokharel, Rakesh Ghimire, Pratik Lamichhane

**Affiliations:** ^1^Maharajgunj Medical Campus, Institute of Medicine, Kathmandu, Nepal; ^2^Department of Clinical Pharmacology, Maharajgunj Medical Campus, Institute of Medicine, Kathmandu, Nepal

## Abstract

Visceral leishmaniasis, also known as kala-azar is one of the most commonly neglected tropical diseases affecting a large number of rural and resource-limited people in South Asia, Africa, and South America. Paromomycin, an aminoglycoside drug, is frequently used for the treatment of visceral leishmaniasis. Despite limited therapies for visceral leishmaniasis and emerging drug resistance, a proper review on the action of paromomycin for kala-azar is lacking. This systematic review aims to look for the efficacy and safety aspects of paromomycin for the treatment of visceral leishmaniasis.

## 1. Introduction

Visceral leishmaniasis (VL) commonly known as kala-azar is characterized by fever, hepatosplenomegaly, and pancytopenia [1]. The disease is primarily caused by protozoan parasite of species *Leishmania donovani* and *Leishmania infantum*, transmitted to humans by the bite of female sand fly, *Phlebotomine*. Leishmaniasis is considered one of the most neglected diseases due to its strong association with poverty and limited resources invested in new tools and technologies for the diagnosis, treatment, and control [2, 3]. The recent 2017 Global Burden of Disease Study estimated that Neglected Tropical Diseases (NTDs) were responsible for 62 million Disability-Adjusted Life Years (DALYs), with 774,000 DALYs from leishmaniasis [4]. In 2015, VL contributed 97% of the total DALYs for the leishmaniases [5], ranking it as the second leading cause of parasitic deaths after malaria [6].

Visceral leishmaniasis is endemic in 79 countries, mainly in the regions of the Indian subcontinent, East Africa, and South America. According to the World Health Organization (WHO), an estimated 50,000 to 90,000 new cases of VL occur worldwide annually [7]. As per the WHO database till January 2021, more than 90% of global VL cases were reported from eight countries: Brazil, Eritrea, Ethiopia, India, Kenya, Somalia, South Sudan, and Sudan [8]. Frequent epidemics of VL occur in East Africa and South Asia. In South America, 97% of the VL cases concentrated in Brazil. However, there has been a geographic expansion of the disease into neighboring countries leading to rise in imported cases in Argentina, Colombia, and Uruguay [9].

Treatment options for VL are limited to few groups of drugs. In the past decades, pentavalent antimony compounds were the first-line therapy for VL, but due to their innate toxicity [10, 11] and frequent parasitic resistance [12], newer drugs such as amphotericin B, miltefosine, and paromomycin got approval for the treatment of VL. In cases of resistance to antimony compounds, amphotericin B (AmB) is preferred for treatment. Amphotericin B is an expensive drug requiring hospitalization and close monitoring for weeks. The liposomal form of amphotericin B has a shorter course of treatment but is even more expensive with nearly thirty times the cost of conventional therapy for VL [13, 14]. Miltefosine despite being oral therapy for VL is said to have potential teratogenic effects and frequent gastrointestinal side effects [15].

**Figure 1:**
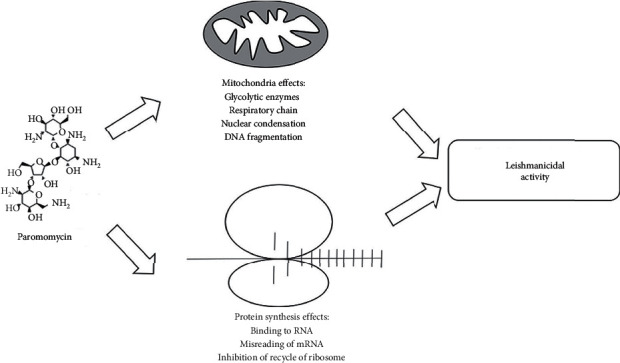
Mechanism showing leishmanicidal activity of paromomycin.

Paromomycin (PM) also known as monomycin or aminosidine is an aminoglycoside group of broad-spectrum antibiotics [16] with activity against *Leishmania*. The mechanism of PM against *Leishmania* is not completely understood yet. However, it is proposed that PM causes inhibition of translocation and recycling of ribosome subunits as well as modification in mitochondrial membrane potential leading to protein synthesis inhibition in *Leishmania* parasite [17]. This mechanism of action of PM against *Leishmania* is shown in Figure 1.

Especially, the intramuscular paromomycin at various doses is used for the treatment of VL. Due to the increasing importance of paromomycin as a therapeutic option for the treatment of VL for the last three decades and increasing trends of drug-resistant parasite, we aimed to conduct a systematic review to look for the efficacy of injectable paromomycin and to review its safety aspects.

## 2. Methods

### 2.1. Literature Search Strategy

A systematic literature search of databases such as PubMed and Google Scholar was conducted to identify all the relevant published articles from 1990 till April 10 2021. PubMed search was conducted using MeSH terms and keywords “Paromomycin,” “Monomycin,” “Aminosidine,” “Visceral Leishmaniasis,” “Kala azar,” and “Black Fever” using suitable Boolean operators. Similarly, Google Scholar search was conducted using advanced search with similar keywords and suitable combinations. Authors of some studies were contacted via e-mail and ResearchGate for retrieval of full texts and clarification of doubts wherever required.

### 2.2. Study Selection

Literature search was performed according to the inclusion criteria. After reading all abstracts, key articles were identified by consensus. Full articles were obtained for all studies meeting the inclusion criteria for further assessment. Bibliographies of selected articles were also searched to identify relevant studies. The final list of included studies had the concurrence of all authors.

### 2.3. Eligibility Criteria

#### 2.3.1. Inclusion Criteria

All articles published in English language between 1990 and 2021 in any setting with an aim of finding the efficacy and safety issues of injectable paromomycin for visceral leishmaniasis regardless of the *Leishmania* species whether *L*. *donovani* or *L*. *infantum*.

#### 2.3.2. Exclusion Criteria

  Studies aimed at finding efficacy of combination of paromomycin with any other drug used for treatment of VL.
  Efficacy and safety studies of paromomycin in animals.
  Paromomycin studies on VL considering other than efficacy and safety issues.
  Case reports, review articles, conference papers, letter to the editor, and articles published in languages other than in English.
  Full texts not accessible/irretrievable.

**Figure 2:**
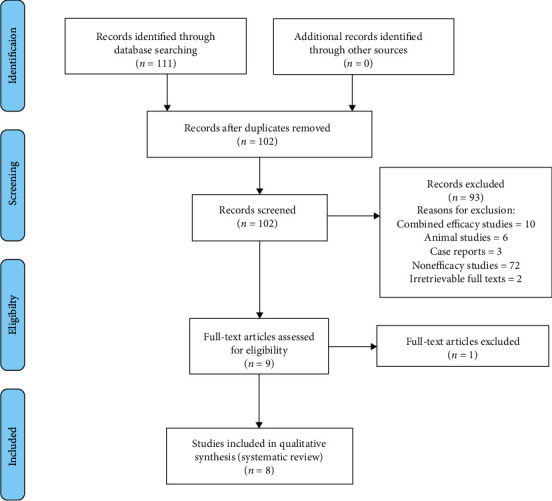
PRISMA flow diagram for study selection for the systematic review.

The systematic review was guided by the Preferred Reporting Items for Systematic Reviews and Meta Analyses (PRISMA) guidelines. The PRISMA diagram detailing the selection process is shown in Figure 2.

### 2.4. Data Collection and Data Items

Studies obtained from the electronic databases, supplementary sources, and manual searching were exported to EndNote reference software version 20 (Thomson Reuters, Stamford, CT, USA) in the compatible formats. Duplicate articles were screened first by EndNote and then manually. Duplicates were then recorded and removed. For multiple publications of the same data in more than one journal, the most inclusive, comprehensive studies, with larger sample size, and the most recent ones were considered.

The data items extracted from each study included author, journal and year of publication, country/place of study, study design, study population, age group, inclusion and exclusion criteria, dose and duration of paromomycin, outcome of treatment (efficacy), and adverse effects (safety issues).

## 3. Results

### 3.1. Study Selection

The initial electronic search identified 111 articles. After adjustment of duplicates, 102 articles remained. Of these, 93 articles were excluded after reading their titles and abstracts as they did not meet the inclusion criteria. Nine full-text articles were reviewed for eligibility, and finally, 8 were included for systematic review.

### 3.2. Study Characteristics

**Figure 3:**
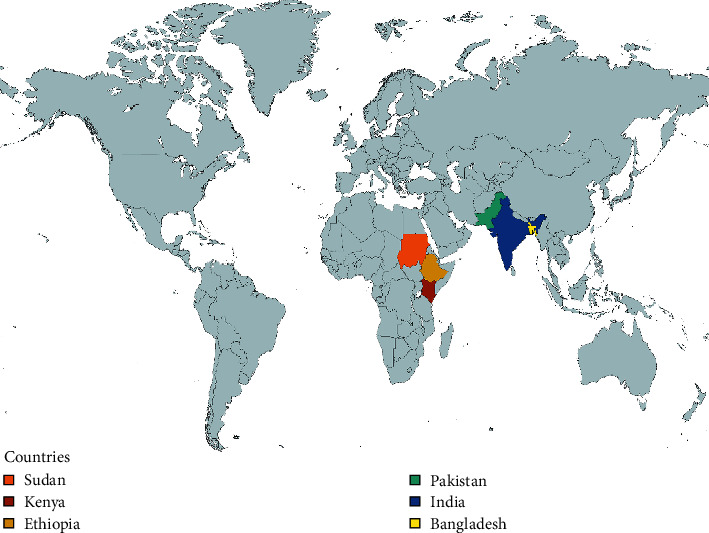
Countries included in the study.

Altogether 8 articles were included in this review, out of which 7 were randomized controlled trials and one was a cross-sectional study (Hassan et al.). The studies covered a total of 2225 participants, out of which 1725 participants were treated with paromomycin. The study population ranged from 42 (Musa et al.) to 666 [1]. The studies were conducted in 7 different countries, one from Pakistan, one from East Africa (Sudan, Ethiopia, and Kenya), one from Bangladesh, one from Sudan, and four from India. This is illustrated in Figure 3. The year of publication of studies ranged from 1995 [18] to 2015 [19]. Visceral leishmaniasis was diagnosed in the studies using standard methods such as demonstration of parasites in bone marrow smear or rapid diagnostic test such as rk37. All studies had utilized microscopic examination of bone marrow or spleen aspirates to diagnose the patients, whereas, rk39 tests were employed by two studies (Jamil et al., Sinha et al.) to further confirm the diagnosis. None of the studies have clearly described the type of *Leishmania* species causing the disease among the study subjects. A detailed description of the characteristics of individual studies is shown in Table 1.

### 3.3. Comparison of Treatment

The study conducted by Hassan et al. used 15 mg/kg/day im paromomycin for 4 weeks. Hailu et al. used 15 mg/kg/day im for 21 days. Jamil et al., Sinha et al., and Sundar et al. [1] used paromomycin dosed of 11 mg/kg/day im for 21 days. The study conducted by Jha et al. used 12 mg, 16 mg, and 20 mg per kg body weight for 21 consecutive days. Similarly, the study by Sundar et al. [1] used 11 mg/kg/day for 14 days in one group and the same dose for 21 days in another group.

### 3.4. Comparison of Outcomes

The outcomes of the studies were the efficacy and safety. Efficacy was evaluated by initial clinical response which was defined as resolution of fever and reduction of splenomegaly at end of treatment. Final clinical response was defined as the absence of new clinical signs and symptoms of VL 6 months after end of treatment or absence of *Leishmania* parasites in the tissue aspirates at the end of 6 months. Safety issues were evaluated by the adverse events which occurred over the period of observation. Nonserious Adverse Events (AEs) were classified according to MedDRA, version 10, and defined as treatment emergent (TEAE) if onset was between the first day of treatment and 30 days after treatment. Severe or life-threatening AEs are grade 3 or 4 adverse events according to Cancer Therapy Evaluation Program-Common Terminology Criteria for Adverse Events (CTEP-CTCAE) definition.

### 3.5. Efficacy/Cure

The efficacy of injectable paromomycin was variable depending upon the dose and duration of treatment. The initial efficacy was 100% in the study conducted by Hassan et al. using 15 mg/kg/day im paromomycin for 4 weeks and 67.4% in the study of Hailu et al. using 15 mg/kg/day im paromomycin for 21 days. In rest of the studies, the initial clinical response ranged from 85.7% in the study of Musa et al. conducted using 20 mg/kg/day for 21 days group to 98.6% in the study of Sundar et al.[1] conducted using paromomycin dosed 11 mg/kg/day for 21 days.

The final efficacy was highest of 97% in the study conducted by Jha et al. in the group of paromomycin dosed 20 mg/kg/day for 21 days and lowest of 63.8% in the East African study conducted by Hailu et al. using 15 mg/kg/day im paromomycin for 21 days. In the study of Hassan et al., the efficacy at the end of six months was not evaluated, but there were no relapses in follow-up to 1 year. A detailed description of the dose- and duration-based initial and final efficacy is shown in Table 2.

### 3.6. Safety Issues on Paromomycin

#### 3.6.1. Adverse Events during Treatment with Paromomycin

All the studies in this review had collected data on Adverse Events (AEs) during treatment with paromomycin. Seven of the eight studies reported at least one adverse event among their participants. The total number of Treatment Emergent Adverse Events (TEAEs) among the studies ranges from 3 to 561. Four studies had reported severe or life-threatening AEs which have affected 63 subjects in total. Six of the studies in this review had reported subjects being discontinued from their study due to TEAEs. Furthermore, four studies had reported 40 Severe Adverse Events (SAEs) in total. A total of 6 deaths of subjects during study duration had been reported by four studies. Deaths in three studies had been reported as unrelated to the study drug [1, 19].  Table 3 illustrates the number of patients who had different types of AEs during the treatment with paromomycin.

#### 3.6.2. Classification of Nonserious AEs Reported during the Study

Paromomycin was well-tolerated, and the majority of the nonserious AEs reported by the studies were from the investigations. Increase in levels of alanine aminotransferase, aspartate aminotransferase, and blood alkaline phosphatase was commonly reported investigations. General disorders and administration site conditions are the second most common nonserious AEs. Injection site pain was the most frequent AE among general disorders and administration site conditions. Other nonserious AEs, which had been considered related to the paromomycin, are illustrated in Table 4.

## 4. Discussion

The objective of this systematic review was to look for the efficacy of injectable paromomycin for the treatment of visceral leishmaniasis and review its safety factors. The efficacy was studied by looking at the outcome at the end of treatment and cure rates at the end of six months of treatment. Safety aspects were reviewed by looking at the adverse events that happened during the treatment.

In the Indian population, the study conducted by Jha et al. showed a cure rate of 97% with paromomycin (PM) dosed 20 mg/kg/day for 21 days but only 77% cure rate with PM dosed 12 mg/kg/day for 21 consecutive days. However, the recent trials conducted by Sundar et al. and Sinha et al. showed a cure rate of more than 90% with PM dose of 11 mg/kg/day for 21 days. In the only head-to-head trial of AmB deoxycholate and paromomycin conducted by Sundar et al., the authors concluded that the PM was noninferior in achieving definitive cure based on noninferiority testing with a noninferiority margin of 0.1. The percentage difference in achieving definitive cure between the two regimens (4.2%) and the upper bound of the 97.5% CI for this result (6.9%) did not exceed the margin of noninferiority. In both groups, mortality rates were less than one percent [1, 22, 23]. Also, the trial conducted in Bangladesh showed an efficacy of 94.2% with the same dose and duration. Similarly, the study of Pakistan showed excellent efficacy of PM for the treatment of VL in children.

In the multicenter study conducted in East Africa (Sudan, Kenya, and Ethiopia) in the year 2010, it showed that the overall cure with PM (63.8%) was significantly inferior to that with SSG (92.2%) (difference of 28.5%, 95% CI: 18.8% to 38.8%, *p* < 0.001) [16]. The efficacy of PM varied among centres and was significantly lower in Sudan (14.3% and 46.7%) than in Kenya (80.0%) and Ethiopia (75.0% and 96.6%). This concludes that the efficacy of PM at 15 mg/kg/day for 21 days was inadequate, particularly in Sudan. In the same year, the trial conducted by Musa et al. in Sudan with 20 mg/kg/day for 21 days and 15 mg/kg/day PM for 28 days showed cure rates of 80% and 81%, respectively (95% CI). The cure rates of VL with PM were still lower than those with SSG. As the parasite type whether *L*. *donovani* or *L*. *infantum* was not isolated in the study, we could not speculate the correlation between the *Leishmania* species and low efficacy of the drug in African population. However, the potential causes for the low efficacy in Africa could be host-related factors such as genetic or immunological variability or parasite-related factors such as innate drug resistance and parasite virulence differences. A study conducted by Verrest et al. concluded that differences in paromomycin pharmacokinetics do not explain the geographical variability in efficacy of paromomycin monotherapy among Eastern African and Indian VL patients [24]. So, we do not recommend PM as a first-line drug in Africa instead the drug can be reserved for cases resistant to SSG or places where AmB cannot be afforded. Also, further studies in potential parasite factors and host factors are required to find out the likely causes.

Furthermore, there are no efficacy studies of PM for the treatment of visceral leishmaniasis from the endemic areas of South America till date. Therefore, there is an immediate need for efficacy and safety studies in this region to support evidence-based kala-azar treatment.

Paromomycin is a relatively safe drug with very few serious adverse events and events of death. Even though paromomycin is an aminoglycoside, its well-known side effects such as ototoxicity and nephrotoxicity are reported in few patients of VL. Moreover, a large number of increases in liver enzymes reported by the studies are reversible, and only a few of those patients required discontinuation of therapy. The increase in liver enzymes is considered to be the result of the destruction of parasites in the liver. Parenteral aminoglycosides are rarely associated with the increase in liver enzymes in general [25]. Hearing impairment seen during or at the end of the treatment in the studies resolved with time, and no permanent changes were reported. Hence, periodic audiometric testing should be conducted for the patients receiving treatment with paromomycin. Proper management of general disorders such as pyrexia and nausea along with administration site conditions such as injection pain will increase the overall compliance of the patient to the treatment.

Our study has few limitations. Studies from various kala-azar endemic countries were lacking, so optimal dose of PM for treatment of VL worldwide could not be estimated. Also, we had two irretrievable full texts which could have affected the potential outcomes. Owing to the above limitations, the results of this review should be interpreted with caution.

## 5. Conclusion

Paromomycin can be a drug of choice for treatment of visceral leishmaniasis in the Indian subcontinent and in resource-limited settings where expensive drugs such as L-AmB are not available readily. The optimal dose of paromomycin for treatment of VL in the Indian subcontinent is 11 mg per kg body weight for 21 days. However, in regions of Africa, paromomycin should be reserved for cases where the efficacy of other first-line antileishmanial drugs is limited. Further single and head-to-head trials of paromomycin should be conducted in kala-azar endemic areas to know the exact efficacy and dose of antileishmanial drugs and to eliminate kala-azar as a global health problem.

## Figures and Tables

**Table 1:** Characteristics of the included studies.

Author	Year	Journal	Place/country	Study population	Age group	Study design
Hassan et al. [18]	1995	J Pak Med Assoc	Islamabad Pakistan	PM = 14, SSG = 36	1–5 yrs = 40, <1 yrs = 5, >5 yrs = 5 (mean age = 2.6 yrs)	Cross sectional
Hailu et al. [16]	2010	PLoS Negl Trop Dis	East Africa (Sudan, Ethiopia, and Kenya)	PM = 135, SSG = 135, SSG + PM = 135	4–60 yrs (mean age = 17.8)	Prospective, randomized, open label, 3-arm trial carried out in five centres of East Africa
Jamil et al. [19]	2015	PLoS Negl Trop Dis	Bangladesh	120	5–55 yrs	Phase IIIb open label, multicenter, single-arm trial
Jha et al. [20]	1998	BMJ	Bihar, India	PM 12 mg group = 30, PM 16 mg group = 30, PM 20 mg group = 30, SSG 20 mg group = 30	6–55 yrs	Randomized unblinded controlled trial 4-armed study with 30 patient each for aminosidine dosed 12, 16, or 20 mg/kg/day for 21 days and rest 30 patient for sodium stibogluconate 20 mg/kg/day for 30 days
Musa et al. [21]	2010	PLoS Negl Trop Dis	Sudan	PM 20 mg group = 21, PM 15 mg group = 21	4–60 yrs	Two-armed, randomized open label dose finding phase II study at a single site in Sudan, randomly assigned to 2 groups
Sinha et al. [22]	2011	Journal of Tropical Medicine	Bihar, India	494	2–55 yrs	Phase IV open label trial
Sundar et al. [1]	2007	N. Engl J. Med.	Bihar, India	PM = 501, AmB = 165	5–55 yrs	Open label, prospective, randomized trial comparing paromomycin with amphotericin B (3 : 1 block)
Sundar et al. [23]	2009	Clin Infect Dis	Bihar, India	PM 11 mg for 14 days = 217, PM 11 mg for 21 days = 112	5–55 yrs	Randomized, open label study intended to assess the efficacy and safety of 2 regimens of paromomycin administered intramuscularly

**Table 2:** Efficacy of injectable paromomycin.

Author	Year	Dose and duration	Initial efficacy (at the end of treatment)	Final efficacy (at the end of 6 months)
Hassan et al. [18]	1995	15 mg/kg im daily for 4 wks.	14/14 (100%)	Not assessed; however, there were no relapses in 1-year follow-up.
Hailu et al. [16]	2010	15 mg/kb body wt. im for 21 days	67.4% (Um el Kher, Sudan = 33.3%, Kassab, Sudan = 60%, Kenya = 86.7%, Gondar, Ethiopia = 66.7%, Arba Minch, Ethiopia = 96.7%) (*p* value < 0.001)	63.8% (Um el Kher, Sudan = 14.3%, Kassab, Sudan = 46.7%, Kenya = 80%, Gondar, Ethiopia = 75%, Arba Minch, Ethiopia = 96.6%) (*p* value < 0.001)
Jamil et al. [19]	2015	11 mg/kg BW im once daily for 21 days	98.3%	94.2%
Jha et al. [20]	1998	12, 16, or 20 mg/kg/day for 21 days	12 mg/kg/day group = 90%, 16 mg/kg/day group = 93.3%, 20 mg/kg/day group = 90%	12 mg/kg/day = 77% (*n* = 23), 16 mg/kg/day = 93% (*n* = 28), 20 mg/kg/day = 97% (*n* = 29)
Musa et al. [21]	2010	20 mg/kg/day for 21 days, 15 mg/kg/day for 28 days	20 mg/kg/day group = 85.7% (95% CI), 15 mg/kg/day group = 90.5% (95% CI)	20 mg/kg/day group = 80% (95% CI), 15 mg/kg/day group = 81% (95% CI)
Sinha et al. [22]	2011	11 mg/kg/day for 21 days	99.6% (95% CI)	94.2% (95% CI)
Sundar et al. [1]	2007	11 mg/kg/day for 21 days	98.6%	94.6%
Sundar et al. [23]	2009	Group A = 11 mg/kg/day for 14 days (*n* = 217); group B = 11 mg/kg/day for 21 days (*n* = 112)	Group A = 91.2%, group B = 96.4%	Group A = 82%, group B = 92%

**Table 3:** Number of patients developing AEs during treatment.

Authors	Year	Journal	Number of subjects	Number of deaths	Patient with at least one adverse effect at any time	Patients with treatment-related AE	Patient with severe or life-threatening AE	Discontinued from study due to ADR	Number of SAEs	No. of patients with TEAEs	Total number of TEAEs
Hassan et al. [18]	1995	J Pak Med Assoc	50	0	0	0	0	0	0	0	0
Hailu et al. [16]	2010	PLoS NTD	135	2	77	75	12	2	5	65	112
Jamil et al. [19]	2015	PLoS NTD	119	1	34	31	2	1	4	32	47
Jha et al. [20]	1998	BMJ	90	0	3	3	0	1	0	3	3
Musa et al. [21]	2010	PLoS NTD	42	0	N/A	N/A	0	0	0	N/A	48
Sinha et al. [22]	2011	Journal of Tropical Medicine	494	2	379	320	35	5	13	379	561
Sundar et al. [1]	2007	NEJM	501	2	299	298	14	5	18	299	353
Sundar et al. [23]	2009	Clinical Infectious Diseases	329	0	N/A	N/A	N/A	4	N/A	N/A	283

**Table 4:** Nonserious adverse events reported.

Authors		Ear and labyrinth disorders	GI disorders	General disorders and administration site conditions	Infections and infestations	Musculoskeletal and connective disorders	Investigations	Nervous system disorders	Psychiatric disorders	Respiratory, thoracic and mediastinal disorders	Skin and subcutaneous tissue disorders
Hassan et al. [18]		0	0	0	0	0	0	0	0	0	0
Hailu et al. [16]		0	6	19	8	0	24	5	0	2	1
Jamil et al. [19]		1	6	26	3	1	0	1	1	2	5
Jha et al. [20]		2	1	0	0	0	0	0	0	0	0
Musa et al. [21]	20 mg/kg/day	3	1	14	1	0	0	0	0	0	1
	15 mg/kg/day	4	0	16	1	0	2	0	0	1	4
Sinha et al. [22]		0	0	49	1	0	484	0	0	0	1
Sundar et al. [1]		3	3	284	0	0	43	0	0	0	0
Sundar et al. [23]		0	0	145	0	0	138	0	0	0	0

## Data Availability

No data were used to support the findings of this study.

## Abbreviations

VL: Visceral leishmaniasis
PM: Paromomycin
NTDs: Neglected tropical diseases
DALYs: Disability adjusted life years
WHO: World Health Organization
im: Intramuscular
BW: Body weight
SSG: Sodium stibogluconate
AmB: Amphotericin B
L. AmB: Liposomal amphotericin B
AE: Adverse Event
TEAE: Treatment Emergent Adverse Event
SAE: Serious Adverse Event
ADR: Adverse drug reaction
CTEP-CTCAE: Cancer Therapy Evaluation Program-Common Terminology Criteria for Adverse Events
N/A: Not available.
